# Indigestion Due to Nervous Influences

**Published:** 1881-06

**Authors:** Charles R. Crandall

**Affiliations:** Philadelphia


### INDIGESTION DUE TO NERVOUS INFLUENCES.

By CHARLES R. CRANDALL, M.D., Philadelphia.

It has long been noted that certain forms of indigestion were dependent upon, or associated with, morbid states of the nervous system. If it was possible to outline such cases in all instances, and say accurately where purely nervous influences began and ended, much would be accomplished toward simplifying a most obscure class of affections. As it is, however, more or less inference, and many facts regarding the development and continuance of a morbid state of the digestion, must be drawn from such habits of life and such symptoms as are associated with expressions of marked nervous phenomena.

That hereditary influences of a nervous character, antagonistic to healthy digestion, exist, there can be no doubt. Children born of nervous and dyspeptic parents are undoubtedly more or less congenitally liable to attacks of indigestion, which are of a purely nervous origin. Such children, during infancy, are especially prone to attacks of vomiting and diarrhœa, from slight improprieties in diet, from the effects of teething, and from changes in the weather. Upon reaching childhood this class are usually excitable, emotional, poorly nourished, and suffer from indigestion whenever depressed from exposure, from overplay or juvenile dissipation. At time of puberty and subsequently, children thus nervously endowed invariably suffer from habitual indigestion, mal-nutrition, chorea, headache, neuralgia, and are easily depressed by excesses of any kind which tend to exalt or exhaust the nervous system. They seem wanting in nervous stamina, in vitality and endurance, and whenever deranged from any cause which makes a powerful impression upon the nervous system, show such evidences of disordered digestion as sick headache, vomiting, loss of appetite, constipation or diarrhœa. Their natural weakness appears to be in the organs of digestion.

As a second cause, tending to originate indigestion by means of impression upon the nervous system, may be mentioned weather and the climatic changes. The marked effect of changes in the weather upon infants is well known. Very frequently they perish in a few days, from deranged digestion induced primarily by a change in the weather from a cool temperature to a hot. murky state.

Adults, not organically diseased, but of moderate or impaired vitality, emigrating from a cool to a warm, prostrating climate, often suffer great nervous depression from prolonged heat. Their symptoms will point chiefly to impaired nutrition, and they generally complain of debility, "bilousness," loss of appetite, constipation or diarrhœa. Indeed, this class of persons, having been stimulated and held up by the cool air of their native heath, invariably fail, and often die, when exposed to the depressing effects of extreme thermometric and barometric changes. Their general condition not being healthy and vigorous, they are unable to withstand the increased waste of moisture, fat and musculine, which is so strikingly shown in many persons by a failure of bodily weight, muscular strength and nervous energy.

Perhaps, the most powerful nervous influence which tends to cause poor digestion is predisposed by sex. High authorities estimate that females are ten times more liable to nervous disorders than males. By nature they are less rugged, more emotional, and more highly organized, and hence are more easily influenced by whatever affects the nervous system. Furthermore, they are naturally and frequently liable to peculiar disturbance resulting from the functions of generation, which also renders them additionally susceptible to extraneous influences. As an outgrowth of such natural state, it is a very common thing to find among this class disorders which are purely nervous in character. There will frequently be evidence of hysteria, anæmia, mental excitement, some form of uterine or ovarian disorder, and then, superadded to all, marked derangement of the functions of digestion. When symptoms of indigestion do exist, they are usually pain in the stomach and bowels, vomiting, flatulence, and habitual constipation, all of which are more or less characteristic of nervous phenomena.

Another origin of disturbed digestion is the powerful influence of intense mental operations. It is a fact, that is known alike to the laity and the profession, that in many instances digestion is largely dependent upon the state of the mind. With very many persons, whatever tends to make a profound mental impression, other than than that of an agreeable nature, tends also to either retard, excite or suspend digestion. Grief, excessive care, constant anxiety, business misfortunes and surprises, not infrequently destroy appetite at once and cause attacks of indigestion, sick headache or diarrhœa. These attacks are usually characterized by prostration, dry mouth, coated tongue, nausea, vomiting and loathing of food. In such instances the nervous system has been shocked and depressed, and, as a result, there is almost a complete suspension of the functions of organic life. Of the immediate exciting causes, sudden emotions, especially of a depressing kind, are among those most frequently cited as having given rise to these disorders; but a similar effect may be produced by moral affections of the same character acting through a longer period. The influence exerted by painful emotion, not only in arresting digestion, but in producing painful sensations in the epigastrium, is well known, and the effects are greatly heightened in case of patients whose nervous susceptibility is more than usually prominent (Fox). An arrest of intestinal digestion may take place from causes other than errors of diet, and give rise to diarrhœa. A strong mental emotion may have this effect. I was present at an operation for hernia, when the surgeon, from the mental anxiety incident to his sense of responsibility, was obliged to relinquish the scalpel and precipitately retire to evacuate the bowels. A gentleman in business receiving, suddenly, unexpected information which led him to know that he was bankrupt, was immediately seized with diarrhœa (Flint). Thus it will be seen how powerful mental impressions, acting through the sympathetic system, may, in some form, completely "upset" digestion; and it is only reasonable to suppose that continuous mental excitement or depression would sooner or later establish a chronic morbid condition.

As a cause of great importance, giving rise to marked manifestations of nervous influence upon the organs of digestion, is hysteria. In this disease, which is supposed to be due largely to exhaustion and mal-nutrition of the nervous centres, there is a tendency to more or less pain, which is almost invariably referred to the stomach and intestines. Out of three hundred and fifty-eight cases analyzed by Briquet, one hundred and eighty-seven had both pain and derangement of the digestive functions. The nervous manifestations in this peculiar disease are many and well known. Among the symptoms, however, usually referred to the organs of digestion, are pain, vomiting, flatulence, acidity and spasm. Indeed, the phenomena may extend to the bowels, giving rise to intestinal indigestion, constipation, abdominal rigidity and phantom tumors. Hence, in a great percentage of all the cases of hysteria there are marked and characteristic nervous influences which tend to produce disturbances of the process of digestion. Most hysterical patients are out of health, many of them being weak and anæmic. It is a remarkable fact, however, that even when they eat a very small amount, nutrition often does not seem to fail. Among the numerous symptoms complained of in different cases may be mentioned *digestive disturbances*, especially flatulence, copious eructations, cardialgia, depraved appetite, fullness after food, obstinate constipation, intestinal colic or gastralgia (Roberts).

Nervous disorders referable to the function of digestion frequently result from an immoderate use of tea, coffee, tobacco and alcohol. In moderate quantities, tea, as a rule, is a very grateful beverage to most persons, and is well borne by the system. When used in excess, however, and especially by persons of susceptible temperament, it causes marked derangements of the nervous system. It gives rise to a peculiar exalted nervous condition, generally termed "nervousness," which is characterized by pallor, wakefulness, impaired appetite and constipation.

Coffee, while as a rule, one of the healthiest of beverages nutritious and well tolerated, may, when taken in excessive amounts, especially by those whose digestion is not in healthy activity, produce distressing evidences of indigestion. Under such circumstances it will cause marked nervous manifestations, with which will be associated characteristic symptoms of indigestion. Hence, it is not uncommon to meet coffee drinkers who suffer from trembling of the hands and limbs, headache, giddiness, irritable heart, pain in the stomach, acidity, flatulence, "biliousness," "belching" and constipation. Simple loss of functional power is also produced by causes immediately affecting the stomach, such as excess of fluids taken at meals, especially when drank warm, or by abuse of narcotics. and of tea and coffee (Fox).

The action of tobacco upon the system is several fold and powerful. That it frequently tends to impair digestion all authorities bear uniform testimony. It may influence digestion by acting as an irritant to the mucous membrane, or it may act as a depressant upon the nerve centres. While well tolerated by the majority of those who become habituated to it, there are, nevertheless, many who are injured by even the most moderate indulgence. Among the latter class it tends to lessen the appetite, waste the saliva, coat the tongue, and so irritate the mucous membrane of the stomach and intestines as to cause chronic indigestion. With these symptoms are also associated its well known action upon the nervous system in causing trembling of the limbs, a weak heart, giddiness, and confusion of mind.

Most authorities agree that alcohol, in a very limited amount, favors digestion. It stimulates the gastric glands, causing them to secrete a greater quantity of gastric juice. But when used habitually to any extent, it tends to dilute the gastric juice and it also tends to precipitate the pepsin. The secretions thus modified are to a certain extent destroyed for healthy digestion, and, as a result, an abnormal condition is established. In just what manner alcohol acts upon the stomach is probably not well known, but it is reasonable to believe that it stimulates the nerves distributed to the gastric glands, mucous follicles and muscular layer. In accordance with the well established law, that over stimulation is followed by depression, in time nervous energy is much impaired and consequently digestion suffers. Moreover, when taken in large quantities it tends to depress the nerve centres and suspend the action of the brain, which in turn is followed by a loss or suspension of nerve muscular power. With nerve and muscular power thus suspended, the action of the stomach, being dependent upon both, is disabled, and digestion necessarily fails. When such a state of affairs is once establised there will also be witnessed the familiar nervous symptoms common to excessive use of alcohol, namely, capricious appetite, uneasiness and pain after meals, morning vomiting, internal heat, craving for drinks and constipation.

Nervous exhaustion is another cause of marked and persistent disturbance of digestion. In the common strife for wealth, fame, fashion and intellectual excellence, together with the tendency to indulge in excess, thousands of persons exhaust their nervous energy. In addition to the wear and tear incident to such a strife, there is also a tendency to goad the nervous system with stimulants, and burden the mind with the depressing influence of fear and anxiety that the object of immense effort will not be attained.

Thus, in a manner both common and disastrous, nervous energy is exhausted and the power necessary to carry on the functions of organic life is largely consumed.

The deleterious effects of such profound and lasting nervous influences upon the functions of digestion cannot be over estimated. Hence, indigestion is one of the most marked symptoms of the morbid condition now recognized as neurasthenia. From want of nerve force, the organs of digestion are no longer able to act, and, therefore, there arise successively all the characteristic evidences of general torpidity of the alimentary tract. The stomach being debilitated, digestion will be slow, causing gastric catarrh and the usual associated conditions of fermative changes, acidity, flatulence, and pain. The intestines being also wanting in nervous and muscular power, there will be neither healthy secretions, absorption or peristaltic action, and, consequently, the most persistent and obstinate constipation will exist Of the special determining causes, *exhaustion* plays the most prominent part, and when combined with other depressing influences, and particularly those of a moral character and operating through the nervous system, such as grief, fear, anxiety, or severe intellectual effort it is an almost unfailing source of perversion of the functions of the stomach, which can only be referred to disturbed innervation. These, however, may originate under almost any circumstances of impaired vitality or of diminished constitutional power (Fox).

In addition to the above-mentioned causes which tend to produce indigestion by impairment of the nervous system, it will be proper to allude briefly to three internal, or centric, morbid conditions, which have a similar effect. These to be referred to are common and highly important to be borne in mind when inquiry is being made into the causation of indigestion.

Accepting the modern view of eminent gynæcologists, that chlorosis, "the green sickness," is "probably a neurosis of the ganglionic system of nerves," a cause is recognized for a marked disturbance of digestion, the origin of which is in the nerve centres. In many instances it is difficult to differentiate chlorosis from anæmia, but the former usually occurring among girls at the time of puberty, without there having existed any of the well recognized causes of anæmia, makes it reasonable to accept the theory that the disease is "symptomatic of functional disease in the sympathetic system of nerves." To state this view in other words: at the critical age of puberty, when a series of important and peculiar changes are being effected through the instrumentality of the sympathetic system of nerves, the system seems, in the female, to be liable to a morbid influence, which, in a great degree, paralyzes it and impairs its functions. Sadness, nervousness, and irrascibility mark its onset; then neuralgia develops in the limbs, the head, and the viscera; the appetite is impaired; digestion becomes weak, and dyspepsia, flatulence and depraved tastes are encountered. The girl craves the most unpalatable and innutricious substances, as, for example, chalk, clay, slate, and other articles of an alkaline nature; while, at other times, the taste prompts her to consume acids, vinegar, lemon juice, pickled vegetables, etc. Usually, the process of blood-making is soon disordered, and anæmia sets in, coincidently with amenorrhœa, constipation, palpitation of the heart, sensitiveness along the spine, distress in the solar plexus of nerves, coldness of the hands and feet, and irregular and excessive flushing of the face (Thomas). Thus, it appears that one of the most aggravated expressions of indigestion and malnutrition, peculiar to young females, in all probability has its seat in, and exerts its influence through, the sympathetic system.

The complex nervous influence exerted upon the functions of digestion by a morbid condition of the uterus is one of the most ancient observations in medical practice. A diseased state, or a displacement, of this important organ sooner or later makes a decided impression upon the nervous system, and ultimately involves the general health. Likewise, ovarian disorders give rise to nervous disturbances, which, in time, are reflected throughout the entire system, imparing the functions of important organs. It is probably safe to say that no other portion of the system suffers so much from nervous influences thus reflected, as do the stomach and intestines. As regards the stomach, when under such influences, its condition is often characterized by the most severe symptoms of functional disorder. Invariably there is either pain, persistent nausea, vomiting, pyrosis, loss of appetite, atonic dyspepsia and tenderness upon pressure. The intestines are also thrown into a morbid condition by irritation thus reflected, and, as a consequence, the patient suffers from distention due to accumulation of gases, as well as from constipation of the most persistent and obstinate character. To these brief allusions may well be added the significant words of a high authority: "With the exception of the last named state, which is, however, far more frequently associated with disorders of the stomach in weakly than in strong, healthy patients, there are few of the uterine derangements here enumerated which are not more or less associated with an impairment of the general nutrition. The majority also appear to be more truly connected, either as a cause or effect, with the primary disorder, than to arise wholly through the disordered digestion which is frequently the last in the series; while with respect to frequency, though not perhaps to severity, leucorrhœa and disordered menstruation hold the foremost rank among this class of causative conditions" (Fox).

Spinal irritation, or anæmia of the posterior columns of the cord, is another morbid condition of the nervous system which exerts a marked influence upon digestion. While sex, as in the female, seems to be the chief predisposing cause of spinal irritation, it is not improbable that in some instances the disease is due to depraved digestion and malnutrition of the nerve centres. But in any event, the seat of the disease is clearly traceable to the posterior columns, and manifests itself chiefly through the sensory nerves. The symptoms are of a character that locate the affection at the origin of the nerves of sensation, although the post mortem evidences are only vaguely confimatory. According to the law, "that irritation at a nerve centre induces pain at points in which the nerve arising from that centre end," when the cervical and dorsal nerves are involved at their origin there will be disturbance in the region where the nerves are distributed. Hence, in a record of one hundred and forty-eight cases nausea and vomiting appeared to have more relation to tenderness of the cervical spine, and pain of stomach to tenderness of dorsal; but that, when there was soreness of both, nausea or vomiting was still more frequent, and pain of the stomach scarcely ever absent (Griffin). And again, in one hundred and fifty-six recorded cases an analysis showed that the dorsal region of the spine was tender in one hundred and sixteen. In thirty-seven of these it was conjoined with cervical, in nineteen with lumbar tenderness, and in fifteen it was affected with the whole spine, leaving forty-five uncomplicated cases. The most prominent symptoms in these cases were connected with the viscera, the stomach being the organ commonly involved. Thus, gastralgia was present in every case, nausea and vomiting in nine cases, pyrosis in three, gastric flatulence in forty, and acidity, as evidenced by heartburn, in twenty-six (Hammond).

Much more could be said in illustration of the place and power of nervous influences in the causation of indiges ion. Furthermore, it would have been interesting to go into the pathology and the treatment of this line of cases, but space forbade. Enough has been said, however, to again call attention to certain manifestations of indigestion dependent upon or associated with, marked disturbances of the nervous system, and, therefore, the object of this paper has been accomplished. The individual experience of nearly every practitioner, as well as many high authorities not herein referred to, would afford valuable confirmation to every thing to which an allusion has been made.

The observations and quotations set forth in this article are neither new nor novel, but simply tend to reaffirm the supreme importance of looking beyond the diet, the stomach and bowels, before making up a diagnosis of indigestion. The practical point in the treatment of most forms of indigestion is to seek out and correct the cause. This can only be done by bearing in mind that there are a multitude of causes, and that many of them are only discoverable to him who makes diligent searches in the labyrinths of obscurity.

Obligations are confessed to: Fox, Diseases of the Stomach; Habershon, Diseases of the Alimentary Canal; Flint, Practice of Medicine; Roberts, Theory and Practice of Medicine; Thomas, Diseases of Women; Hammond, Diseases of the Nervous System; DaCosta, Medical Diagnosis.

